# Investigation of antibacterial and anticancer effects of novel niosomal formulated Persian Gulf Sea cucumber extracts

**DOI:** 10.1016/j.heliyon.2023.e14149

**Published:** 2023-02-28

**Authors:** Tohid Piri-Gharaghie, Ghazal Ghajari, Maryam Hassanpoor, Neda Jegargoshe-Shirin, Mona Soosanirad, Shahoo Khayati, Ali Farhadi-Biregani, Amir Mirzaei

**Affiliations:** a Biotechnology Research Center, Faculty of Biotechnology, Shahrekord Branch, Islamic Azad University, Shahrekord, Iran; b Biotechnology Research Center, East-Tehran Branch, Islamic Azad University, Tehran, Iran; c Department of Cell and Molecular Biology, Faculty of Biological Sciences, Kharazmi University, Tehran, Iran; d Department of Biotechnology, Faculty of Medicine, Semnan University of Medical Sciences, Semnan, Iran; e Department of Biotechnology, Faculty of Basic Sciences, Damghan Branch, Islamic Azad University, Semnan, Iran; f Department of Cell and Molecular Biology, Factuly of Biological Sciences, Yadegar-e-Imam Khomeini (RAH) Shahr-e-Rey Branch, Islamic Azad University, Shahr-e-Rey, Iran; g Department of Immunology, School of Medicine, Ahvaz Jundishapur University of Medical Sciences, Ahvaz, Iran; h Department of Biology, Faculty of Basic Sciences, Parand Branch, Islamic Azad University, Parand, Iran

**Keywords:** Sea cucumber, Antibacterial, Anticancer, Apoptosis, Gene expression

## Abstract

Pharmaceutical companies worldwide are scrambling to develop new ways to combat cancer and microbiological pathogens. The goal of this research was to investigate the antibacterial, anticancer, and apoptosis effects of novel niosomal formulated Persian Gulf Sea cucumber extracts (SCEs). Sea cucumber methanolic extracts were prepared and encapsulated in niosome nanoparticles using thin-film hydration. The compound was made up of Span 60 and Tween 60 blended with cholesterol in a 3:3:4 M ratios. Characterization of niosome-encapsulated SCE evaluated by scanning electron microscopy and transmission electron microscopy. The disk diffusion method and microtiter plates were used to investigate the antimicrobial activity. The effect of niosome-encapsulated SCE on cell proliferation and apoptosis induction was studied using MTT and Annexin V, respectively. The expression of apoptosis-related genes, including *Bax, Fas, Bax, Bak,* and *Bcl2,* was studied using quantitative real-time PCR. Niosome-encapsulated SCE with a size of 80.46 ± 1.31 and an encapsulation efficiency of 79.18 ± 0.23 was formulated. At a concentration of 100 μg/ml, the greatest antimicrobial effect of the niosome-encapsulated SCE was correlated to *Staphylococcus aureus*, with an inhibition zone of 13.16 mm. The findings of the study revealed that all strains were unable to produce biofilms at a concentration of 100 μg/ml niosome-encapsulated SCE (*p* < 0.001). The survival rate of cancer cells after 72 h of exposure to niosome-encapsulated SCE was 40 ± 3.0%. Encapsulated SCE in niosomes inhibited cell progression in MCF-7 cells by increasing G0/G1 and decreasing S phase relative to G2/M phase; as a result, it activated the apoptosis signaling pathway and led to the induction of apoptosis in 69.12 ± 1.2% of tumor cells by increasing the expression of proapoptotic genes (*p* < 0.001). The results indicate that sea cucumber species from the Persian Gulf are a promising source of natural chemicals with antibacterial and anticancer properties, paving the path for novel marine natural products to be discovered. This is the first demonstration that niosome-encapsulated SCE contains antibacterial and anticancer chemicals that, according to their specific characteristics, boost antitumor activity.

## 1 Introduction

Seas are known to be a major supply of metabolites and pharmacological chemicals, as well as a resource of biodiversity and pharmacological action [1]. Antiviral and antibacterial properties have been identified from compounds derived from marine creatures [2]. Marine organisms, especially sea cucumbers, are high in glycosides, particularly triterpene glycosides, which have been shown to have antimicrobial and anticancer properties [3]. There are also significant quantities of lectins, cerebrosides, glycoproteins, sterols, omega-6 fatty acids, and omega-3 acids in these species [4].

Natural compounds in marine organisms can be used as a source of compounds with nutritional, pharmaceutical, and medical applications [5]. Organic chemicals found in marine creatures can be utilized to make substances that have nutritional, pharmacological, and medicinal uses [6]. Moss creatures, Echinodermata, case bearers, fishes, and sponges all have biologically active compounds that may be isolated [7]. Sea cucumber is a unique class of creatures with a body shape unlike any other. These organisms constitute more than 6500 species of marine organisms [9]. Sea cucumbers [Holothuroidea] are among the most unusual members of Echinodermata in terms of both structure and physiology [8,9]. For several years, sea cucumber research has been limited to its physiological and ecological characteristics [10]. Sea cucumbers are now being investigated for antibacterial, antifungal, anticoagulant, antiviral, cytotoxic, hemolytic, and even anti-HIV properties [11]. Sea cucumber extracts have been found to have potent antibacterial properties in previous studies [12,13]. Triterpene glycosides, de-sulfated glycosides, steroid glycosides, polyhydroxy sterols, naphthoquinone pigments, lysozyme, and other precursors are responsible for this antibacterial action [14]. Saponins, chondroitin sulfate, glucose amino glycans, sulfated polysaccharides, and glycoproteins are among the bioactive compounds found [15]. Other characteristics, such as anticancer effects in sea cucumber, may be attributed to lipids and essential amino acids [16].

Cancer is the biggest cause of mortality in developed countries, as snakes demonstrate [17]. In recent decades, researchers have undertaken several attempts to cure this illness [18]. Breast cancer is the fifth most prevalent cause of mortality in women, and it is the most significant and ubiquitous type of cancer in women [19]. Depending on the stage of cancer and the person's health state, surgery, radiation, and chemotherapy are used as standard therapies [20]. They do not, however, have a good therapeutic impact and have many adverse effects. Researchers have undertaken experiments in recent years to study and confirm the cytotoxic impact of several sea cucumber species, and the findings are promising [21]. There was evidence of a significant cytotoxic impact in the investigated species. In certain ways, nanotechnology has achieved considerable progress in medicinal applications, such as novel technological drug delivery methods, as a result of its expanding study in several disciplines of science [22]. In the pharmaceutical sector and therapeutic development, for example, there is much interest in a wide variety of nanostructures [22].

Encapsulation of antimicrobial and anticancer agents in nanocarrier systems is one of the most promising and successful methods for improving antibacterial activity while decreasing adverse effects [23,24]. Niosomes have recently gained popularity as a means of improving selective medication delivery and the therapeutic effectiveness of antimicrobial medicines [[25], [37]]. Niosomes are bilayer structures with nonionic surfactants that make them water soluble and allow them to transport large amounts of medication [26]. Niosomes contain unique properties that might be utilized to encapsulate a variety of medicines [27,[46], [47], [48]]. Researchers have recently focused their attention on the use of niosomes as antibacterial and anticancer nanocarriers [[25], [26], [27]]. Niosomes can also be manufactured for a variety of formulations for usage in various therapeutic settings. For instance, research that looked at revolutionary nano-vesicle-based niosomes for the inhalation therapy of pulmonary disorders finished its Phase I trial in 2017. This research introduces the structure, components, and formulation techniques of niosomes and discusses their prospective clinical uses based on these advancements and the benefits of niosomes [53,54]. As a result, sea cucumber extract was chosen as a powerful antibacterial and anticancer agent in this investigation. In reality, the goal of this research was to create a niosome-encapsulated sea cucumber extract with enhanced antibacterial and anticancer efficacy against MRSA *Staphylococcus aureus* and MCF-7 cell lines.

## 2 Materials and methods

### 2.1 Collection of sea cucumbers

Sea cucumber was a gift from Haj Atieh. Sea cucumbers weighed between 300 and 700 g. Collection of sea cucumber was done by Haj Atieh from a depth of approximately 10–30 m around Bandar-e Kangan port with a longitude of 52.0645° E and a latitude of 27.8370°N. Harvesting was done according to the rules and regulations of Bushehr Province Fisheries Department. (https://shilatbushehr.ir/rule/).Necessary license for collecting sea cucumber was prepared by Haj Atieh with license number 162390601.

### 2.2 Preparation and fractionation of sea cucumber extract

As a gift from Mr. Haj Atieh, each sea cucumber weighing approximately 550 g was prepared frozen and delivered to the Shahrekord Azad University fisheries lab using dry ice. Sea cucumbers were gathered fresh and treated according to standard techniques for preparing extracts. The animal samples were first washed with distilled water, dissected into 2 cm^3^ parts, snap-frozen in liquid nitrogen, lyophilized for 2 days, crushed using an “A11 basic analytical mill,” and kept at −70 °C for extraction. Every 1 g of powdered sample was regenerated in 10 ml of 80% ethanol, homogenized for 2 min on ice with a professional Tissue-Terror, and subsequently centrifuged at 2500 rpm for 10 min at 4 °C. The supernatant was lyophilized after being filtered via a plastic film of 100 μm. The ethanol-extracted lyophilized substance was resuspended in PBS with 10% dimethyl sulfoxide [DMSO], vortexed, and centrifuged at 13,000 rpm for 10 min. The samples were filtered using 0.2 μm filters, and the resultant extracts, known as sea cucumber extract [SCE], were utilized in the experiment as stated.

### 2.3 Preparation and characterization of niosome-encapsulated SCE

#### 2.3.1 Encapsulation of SCE in niosome

Niosome encapsulation of SCE was conducted through thin-film hydration. The nanoparticle was made up of Span 60 [CAS: 1338-41-6] [Henan Daken Chemical CO., LTD., China] and Tween 60 [CAS: 9005-67-8] [Career Henan Chemical Co, China] blended with cholesterol [CAS: 57-88-5] [Capot Chemical Co., Ltd., China] in a 3:3:4 M ratios and suspended in 10 ml chloroform and methanol mixed in 2:1 ratio. After adding glass beads to the complex, the solvents were dried for 60 min at 60 °C and 120 rpm rotation using a rotary vacuum evaporator [Heidolph, Germany]. To generate a good niosome formulation, dried thin films were hydrated for 60 min at 60 °C at a speed of 120 rpm using a solution of SCE mixed in 10 ml of PBS. The obtained nanoparticle was centrifuged at 4 °C for 30 minto decrease the size of niosomes-SCE, and samples were kept at 4 °C for subsequent studies [49,50].

#### 2.3.2 Niosome-encapsulated SCE morphological characteristics and entrapment efficiency [EE]

Using dynamic light scattering [DLS] and zeta-plas palladium electrodes, the uniform distribution, size distribution, and zeta potential of SCE loaded in niosomes were determined [Brookhaven Instruments Corp., USA]. The typical z diameter and multiple scattering index of niosomes were calculated, as well as their zeta potentials. To create electrical conductivity, niosome-encapsulated SCE particles were coated with a gold layer and analyzed with a MIRA3 field scanning electron microscope [FESEM] [TESCAN, Czech Republic]. A TEM apparatus with a magnification of 60,000× and a voltage of 26 kV was used to examine the morphology of the niosomes. The niosome was produced using a grid with a form covering after being treated with glutaraldehyde and then negatively stained with uranyl acetate. The quantity of noncapsulate SCE [free SCE] in the produced niosomes was measured to determine entrapment efficiency. Niosome-encapsulated SCEs were isolated in a refrigerated centrifuge at 14,000 rpm for 60 min. The EE % was calculated by Eq. (1), and the SCE concentration of the supernatant was determined using an ELISA Reader Stat Fax2100 [Awareness Technology, Ukraine] light absorbance measurement of the supernatant at 276 nm wavelength.

(1) %EE = [[Drug added − Free “unentrapped drug”]/Drug added] × 100

Dialysis was employed to examine SCE's release of the noisome. The dialysis tube was soaked in distilled water for 24 h. Then, 0.5 ml (10 mg) of SCE-loaded noisome was placed in a dialysis bag, and 0.5 ml SCE aqueous solution containing 10 mg SCE was also used as a control sample. Dialysis bags were immersed in conical flasks in 75 ml distilled water and shaken at 50 rpm in a water bath at 37 °C. Five milliliters were withdrawn from the receptor medium at intervals of 1, 2, 4, 6, 12, and 24 h, Aliquots of samples were replaced with a new medium at 37 °C, and imipenem was measured by spectrophotometry at 281 nm. The diffusion profile was determined using various kinetic models. This method was used to monitor the stability of diffusion of various Niosome-encapsulated SCE formulations at intervals of 7, 14, 21, 28, 35, 42, 49, and 56 days for a storage period of two months at 25 °C.

### 2.4 Investigation of the antibacterial and antibiofilm effects of niosome-encapsulated SCE

#### 2.4.1 Microbial strains and antimicrobial assay

The sensitivity of different microorganisms to the obtained niosome-encapsulated SCE was tested. *S. aureus* [ATCC 25923], *Streptococcus mutans* [ATCC25175], *Porphyromonas gingivalis* [ATCC 33277], *Tannerella forsythia* [ATCC 43037] and *Candida albicans* [ATCC 10231] were among the microorganisms supplied by the Iranian Biological Resource Center [http://www.en.ibrc.ir/]. These strains are deemed positive biofilm isolates of their respective species. Antimicrobial content was assessed using an agar well diffusion experiment. A total of 108 colony-forming units [CFU]/ml inoculated ratios of fungi and bacteria were introduced in 6 mm diameter wells of nutrient agar and potato dextrose agar [PDA] medium [NEOGEN, USA] in Petri plates. Employing dimethyl sulfoxid [DMSO] as a solvent, 100 μg/ml niosome-encapsulated SCE was produced. Each plate had 50 μl of niosome-encapsulated SCE introduced into each well and incubated at 37 °C overnight. To determine the antibacterial and antifungal activities of niosome-encapsulated SCE, the average diameter of the inhibitory zones in millimeters was measured. For each substance under investigation, the average of the three replicates was computed. The positive control was ciprofloxacin dissolved in DMSO at 100 μg/cm^3^, while the negative control was sterile DMSO.

#### 2.4.2 Biofilm growth inhibition [% BGI]

The efficacy of niosome-encapsulated SCE biofilm growth suppression by attachment to model biofilms was assessed using the Vyas [2007] technique [27], which involved evaluating the OD630 decrease of biofilms exposed to free SCE and niosome-encapsulated SCE for 2 h. Biofilm-containing wells were treated with 100 μg/ml niosome-encapsulated SCE in a 200 μl PBS solution. Control groups included free SCE and SCE combined with a blank niosome. The liquid components of each well were aspirated, and biofilms were cleaned with ethanol after 2 h of incubation at 37 °C. After that, antibiotic-free culture was introduced to the wells, and the biofilm and aspirate liquid contents were fixed and washed. Biofilms were subsequently dyed with crystal violet, their light absorbance was measured at 630 *λ*_max_ nm wavelength with an ELISA Reader Stat Fax2100 [Awareness Technology, Ukraine], and the amount of biofilm growth inhibition [% BGI] was calculated using Eq. (2).

(2) BGI%={″[OD630×UntreatedBiofilm]−[OD630×TreatedBiofilm]″÷[OD630×UntreatedBiofilm]}×100

### 2.5 Evaluation of the cytotoxic and anticancer activity of niosome-encapsulated SCE

#### 2.5.1 Cell culture and niosome-encapsulated SCE treatment

HUVEC and MCF-7 cell lines were grown in DMEM with 10% FBS [Sigma–Aldrich, USA], 2 mM glutamine, and antibiotics [penicillin G, 60 mg/L; streptomycin, 100 mg/L; and amphotericin B, 50 mg/L] before being exposed to a humid and warm medium [37 °C, 5% CO2, 95%]. The standard solutions [100 mg/ml SCE in DMSO] were added to the medium containing enough volumes to obtain the desired concentrations and then cultivated with cells for 24 h, while the DMSO solution served as a blank reagent, for both niosome-encapsulated SCE and free SCE treatment.

#### 2.5.2 Determination of cell viability

MTT assay was used to assess the vitality of MCF-7 cancer and HUVEC normal cell lines after treatment with niosome-encapsulated SCE and free SCE. In a 24-h incubation period, cells [10000/well] were treated with 0, 100, 150, and 200 μg/ml niosome-encapsulated SCE and free SCE. After that, the cells were washed twice in isotonic phosphate-buffered saline solution [PBS], and 0.5 mg/ml MTT [Thermo Fisher Scientific, United States] was added to each well and kept at 37 °C for 3 h. Formazan crystals were produced and dissolved in 100 μL/well DMSO. After that, an ELISA reader [Organon Teknika, Netherlands] read the absorbance at 570 nm. The amount of toxicity was measured by the equation below:

Cytotoxicity % = 1 − Mean absorbance of toxicant/Mean absorbance of negative control × 100

#### 2.5.3 Apoptosis detection

MCF-7 cells from the niosome-encapsulated SCE group, free SCE group and the blank group were washed twice in PBS and then incubated for 15 min with FITC-Annexin V [Invitrogen TM, United Kingdom]. A CyFlow ML flow cytometer was used to examine apoptosis [PARTEC, GERMANY]. Multi-Cycle AV software was used to examine apoptosis data *[Phoenix Flow Systems*, Biotechnology Company in San Diego, California].

#### 2.5.4 Cell cycle analysis

The niosome-encapsulated SCE group, free SCE group, and blank group of MCF-7 cells were fixed for 24 h in absolute ethanol. Before being stained for 15 min with BD Bioscience Pharmingen's PI/RNase staining buffer, the cells were washed twice in PBS. FACS flow cytometry was used to determine the DNA content of the cell population. FlowJo version 10 software (https://www.bdbiosciences.com/en-eu/products/software/flowjo-v10-software) was used to evaluate cell cycle data [Tree Star, Ashland, OR].

#### 2.5.5 Expression of apoptosis-related genes by quantitative real-time PCR

A quantitative real-time PCR approach with SYBR green detection was used to examine the expression of the proapoptotic genes FAS, BAK, BAX, and P53, as well as the anti-apoptotic genes BCL2 and SURVIVIN. The manufacturer's approach for obtaining RNA was TRIzol [Invitrogen, Carlsbad, CA]. Using the Transcript RT kit [Tiangen Biotech, Beijing, China], first-strand cDNA was created. Quantitative real-time PCR was carried out utilizing specific primers [Table 1] and a SYBR® Premix Ex Taq™ II kit [TaKaRa, Japan] based on the manufacturer's instructions. A Rotor gene 6000 Corbett system was employed for amplification. Thermal cycling conditions were set as follows: an initial activation step for 5 min at 95 °C, followed by 40 cycles of 95 °C for 15 s and 60 °C for 1 min. Standard curves were created using data from serially diluted samples to confirm the reaction efficiencies of each primer set. Each primer set was also subjected to melting curve analysis. To verify the product size, PCR products were electrophoresed on a 1% agarose gel. *GAPDH* was employed to be the control.

**Table 1:** Details of oligonucleotide primers that were used for PCR and real-time PCR.

Target	Primers Name	Sequences 5′→3′	Annealing Temperature [°C]	Product length [bp]
*P53*	P53-F P53-R	TGCGTGTGGAGTATTTGGATGAC CAGTGTGATGATGGTGAGGATGG	64	170
*Fas*	FAS-F FAS-R	CAATTCTGCCATAAGCCCTGTC GTCCTTCATCACACAATCTACATCTTC	64	163
*Bax*	BAX-F BAX -R	AGGTCTTTTTCCGAGTGGCAGC GCGTCCCAAAGTAGGAGAGGAG	65	243
*Bak*	BAK-F BAK-R	CGTTTTTTACCGCCATCAGCAG ATAGCGTCGGTTGATGTCGTCC	66	154
*Bcl-2*	BCL2-F BCL2-R	GACGACTTCTCCCGCCGCTAC CGGTTCAGGTACTCAGTCATCACCAC	65	245
*SURVIVIN*	SURVIVIN-F SURVIVIN-R	AGAACTGGCCCTTGGAGG CTTTTTATGTTCCTCTATGGGGTC	64	170

### 2.6 Statistical analysis

All of the trials were carried out three times. The mean difference between groups was estimated using independent T test or analysis of variance [ANOVA] statistical techniques in the Statistical Package for Social Sciences [SPSS, Inc., Chicago, IL, USA] version 20 (https://www.ibm.com/support/pages/downloading-ibm-spss-statistics-20). GraphPad Prism version (https://www.bioz.com/result/graphpad%20prism%207%200%20software/product/Graph%20Pad%20Software%20Inc) for Windows [GraphPad Software, USA] was used to create the graphs. All P values were less than 0.05, indicating that they were statistically significant.

## 3 Results

### 3.1 Niosome-encapsulated SCE with a uniform spherical structure and low PDI index has high entrapment efficiency

Formulations of niosome-encapsulated SCE (100 μg/ml) were studied morphologically. The chemical composition of extracts of sea cucumber body organs was measured by GC-MS and recorded in Table S1. This formulation had a distinct size, polydispersity index [PDI], and entrapment efficiency [EE]. Dynamic light scattering [DLS] revealed that formulations of niosome-encapsulated SCE had good uniformity [Table 2]. As demonstrated, this formulation of niosome-encapsulated SCE is of a smaller and better size and is associated with surfactant Span60's hydrophile-lipophile balance. Span60 has a hydrophile-lipophile balance of 4.7. Therefore, this nanoparticle formulated with Span60 is 80.46 ± 1.31 nm in size. In addition, the EE content of this formulation was 79.18 ± 0.23. The permeability of SCE in niosomes is directly related to the length of the saturated alkyl chain; thus, longer saturated alkyl chains result in higher permeability. Because Span60 has a long alkyl chain, formulations including it have higher indices, and the EE content is at its highest in this formulation. A lower polydispersity index [PDI] shows a suitable distribution of small nanoparticles, implying that such a formulation is the best since it has the lowest PDI.

**Table 2:** Morphological features of niosome-encapsulated SCE compared to blank niosomes.

Formulations	Polydispersity index [average ± SD]	Zeta Potential [m.v]	Vesicle size [nm] [SEM]	EE [%]
Blank niosome	0.484 ± 0.79	−85.12 ± 1.75	75.32 ± 0.34	–
Niosome-encapsulated SCE	0.187 ± 0.24	−63.19 ± 2.68	80.46 ± 1.31	79.18 ± 0.23

**Fig. 1:**
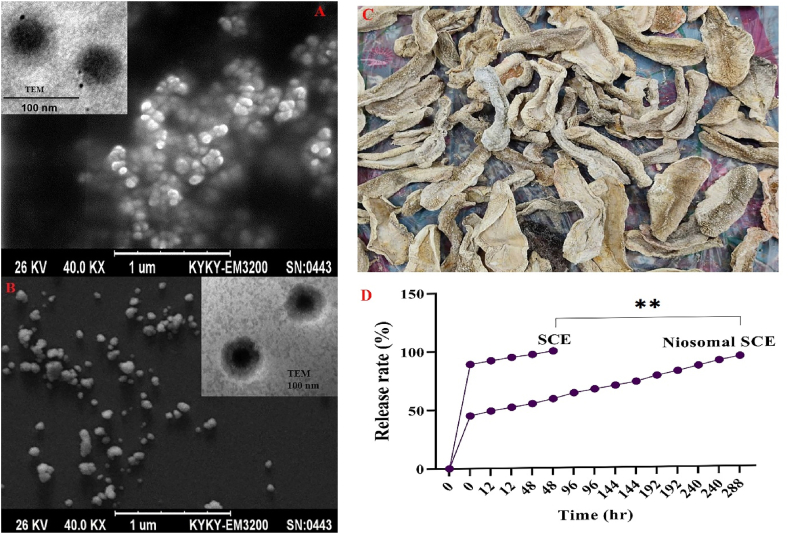
TEM and SEM with a magnification of 60,000× and a voltage of 26 kV were used to examine the morphology of the free niosome [A] and the optimal niosome containing SCE [B]. The particles have a spherical structure, as illustrated in the diagram. [C] Sea cucumber after the drying process. [D] The rate of controlled drug release of Niosome-encapsulated SCE compared to free SCE. **p < 0.01.

Fig. 1 shows that the niosome-encapsulated SCE of this formulation has a homogeneous spherical shape with an average size of 80.46 ± 1.31, indicating that the drug formulation's diameter [100 μg/ml] is acceptable. The particles have a spherical structure, as illustrated in the TEM diagram.

### 3.2 Niosome-encapsulated SCE Increases the antimicrobial and antibiofilm properties of SCE

The outcomes of 100 g/ml methanol extracts of niosome-mixtured SCE, niosome-encapsulated SCE, and free SCE were investigated. *S. aureus* was utilized as a bacterial species for the antibacterial and antifungal assays. The fungus species *C. albicans* was utilized. The niosome-encapsulated SCE had the best impact. It had the greatest inhibitory impact on the strains indicated. At a dosage of 100 μg/ml, the greatest effect of the niosome-encapsulated SCE was linked to the *S. aureus* strain, with an inhibition zone of 23.16 mm, while the inhibitory zone for *C. albicans* was 10.99 mm. The maximum inhibitory effect of the niosome-mixture SCE and free SCE was also on strains of *S. aureus* at a concentration of 100 μg/ml, with inhibition zones of 14.26 and 14.12 mm, respectively, while the maximum inhibitory effect was significant at a concentration of 100 μg/ml of the strain of *C. albicans*, with inhibition zones of 8.40 and 8.09 mm [Table 3].

**Table 3:** Inhibitory effect of different formulations of sea cucumber in comparison with azithromycin [mm] against bacterial and fungal strains.

Strain	Inhibitory effect of Azithromycin [mm]	Inhibitory effect of different Formulation [mm]		
		Niosome-encapsulated SCE [100 μg/ml]	Niosome-mixed SCE [100 μg/ml]	free SCE [100 μg/ml]
Tannerella forsythia	9.79 ± 0.2	19.23 ± 0.21	13.52 ± 0.13	13.43 ± 0.12
Porphyromonas gingivalis	7.15 ± 0.12	16.54 ± 0.17	12.1 ± 0.15	11.87 ± 0.17
Streptococcus mutans	6.97 ± 0.19	18.48 ± 0.15	12.98 ± 0.14	12.64 ± 0.16
*S. aureus*	7.13 ± 0.09	23.16 ± 0.09	14.26 ± 0.13	14.12 ± 0.13
*C. albicans*	2.23 ± 0.14	10.99 ± 0.02	8.40 ± 0.12	8.09 ± 0.14

The microtiter plate technique was utilized to quantitatively examine the antibiofilm effects of all three groups. The findings in this study revealed that all strains were unable to produce biofilms at a concentration of 100 μg/ml niosome-encapsulated SCE, as indicated in Table 4. Other groups, on the other hand, have a comparable capacity to produce biofilms. However, biofilms formed in niosome-mixed SCE groups, and free SCE was weak.

**Table 4:** Quantitative study of antibiofilm properties in strains affected by different formulations.

Strain	Formulation	[μg/ml]	ODC	OD before treatment	OD after treatment	Result	P value
Tannerella forsythia	Niosome-encapsulated SCE	100	0.034	0.18	0.032	Negative Biofilm	0.001
	Niosome-mixed SCE	100	0.035	0.19	0.064	Weak Biofilm	
	free SCE	100	0.031	0.14	0.053	Weak Biofilm	
Porphyromonas gingivalis	Niosome-encapsulated SCE	100	0.042	0.22	0.031	Negative Biofilm	
	Niosome-mixed SCE	100	0.039	0.19	0.071	Weak Biofilm	
	free SCE	100	0.044	0.25	0.083	Weak Biofilm	
Streptococcus mutans	Niosome-encapsulated SCE	100	0.04	0.22	0.035	Negative Biofilm	
	Niosome-mixed SCE	100	0.038	0.18	0.069	Weak Biofilm	
	free SCE	100	0.038	0.20	0.071	Weak Biofilm	
*S. aureus*	Niosome-encapsulated SCE	100	0.036	0.34	0.025	Negative Biofilm	
	Niosome-mixed SCE	100	0.033	0.37	0.061	Weak Biofilm	
	free SCE	100	0.041	0.33	0.073	Weak Biofilm	
C. albicans	Niosome-encapsulated SCE	100	0.045	0.48	0.031	Negative Biofilm	
	Niosome-mixed SCE	100	0.047	0.51	0.059	Weak Biofilm	
	free SCE	100	0.043	0.49	0.074	Weak Biofilm	

OD > 4 × ODc: Strong Biofilm.

ODc < OD ≤ 2 × ODc: Weak Biofilm.

OD ≤ ODc: Negative Biofilm.

**Fig. 2:**
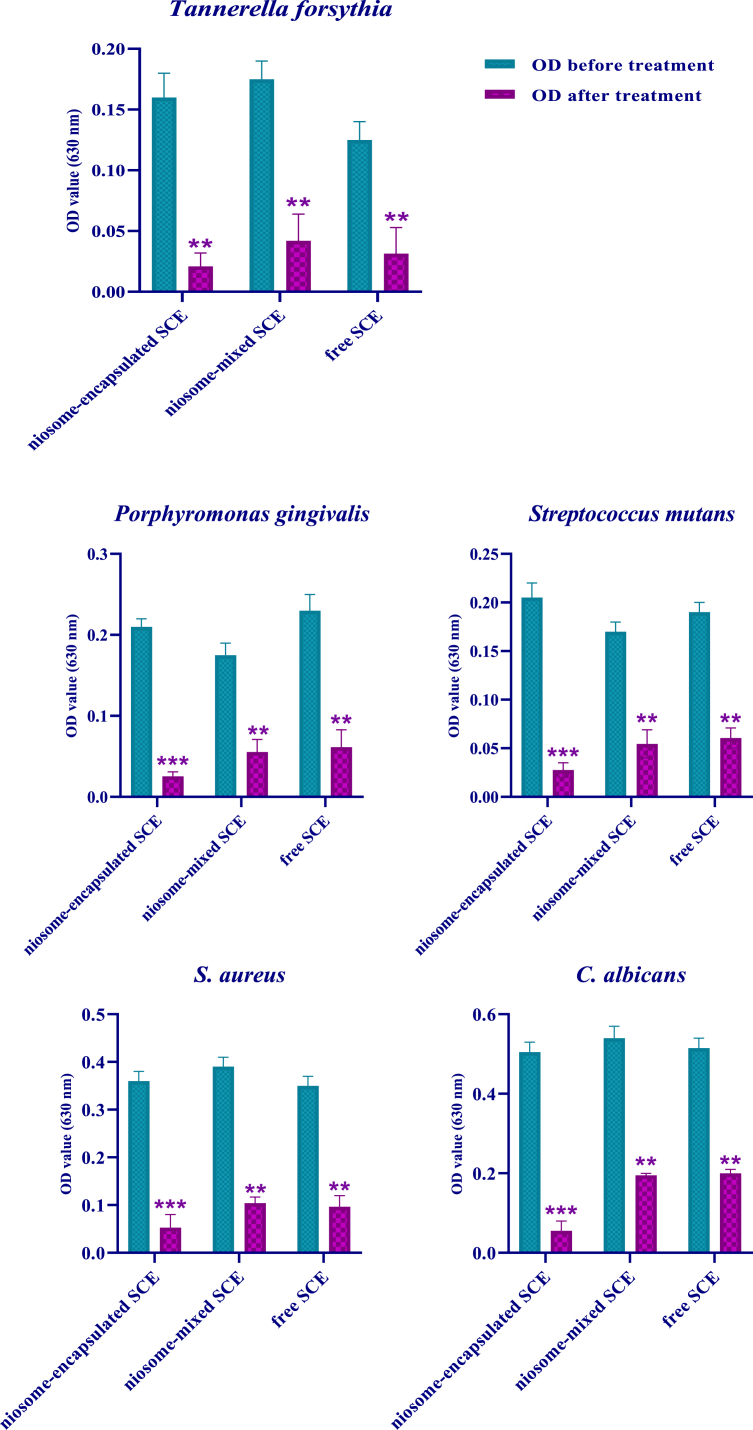
Different formulations of sea cucumber extract at a concentration of 100 μg/ml have strong antibiofilm properties against bacterial and fungal strains. ***P < 0.001, ***P < 0.01.

Fig. 2 shows that following treatment with all three formulations [niosome-mixed SCE, niosome-encapsulated SCE, and free SCE], the light absorption of all strains decreased significantly [*p* < 0.001]. These findings revealed that bacteria and fungi proliferated at a slower rate, resulting in a lower quantity of cell mass and little or minimal biofilm development. Based on these findings, sea cucumber extract exhibits strong antibiofilm effects on both fungal and bacterial strains at a concentration of 100 μg/ml.

### 3.3 Niosome-encapsulated SCE reduces tumor cell proliferation by inducing apoptosis

**Fig. 3:**
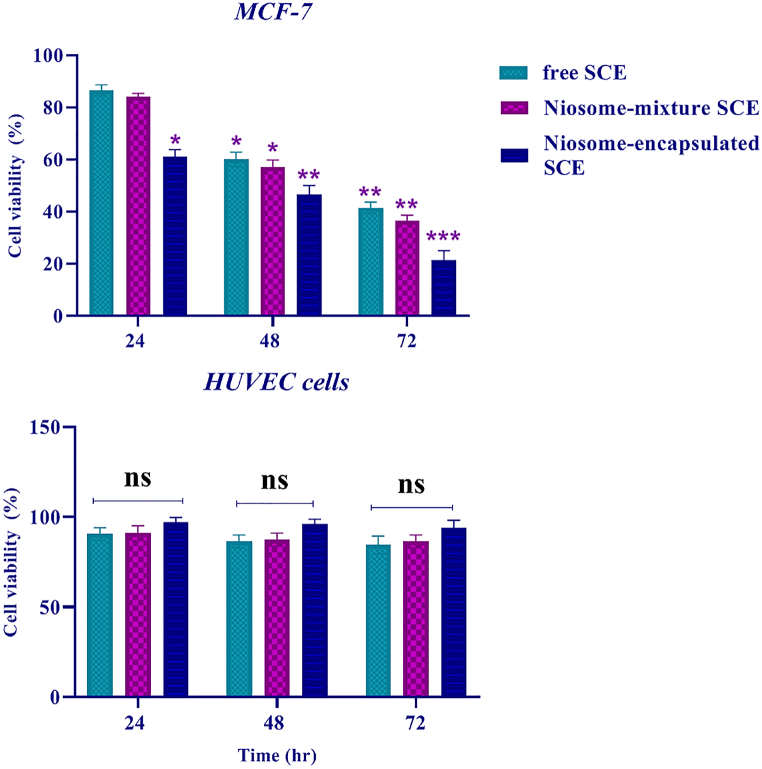
MCF-7 cell survival was considerably reduced in the niosome-encapsulated SCE group compared to the control groups [niosome mixed SCE with and free SCE]. However, sea cucumber extract did not influence the survival rate of normal HUVECs [in all three groups].

*Sea cucumber extract* efficiency in MC7-7 cell lines was assessed. We gathered MCF-7 human breast cancer cell lines into 3 groups [niosome-mixed SCE, niosome-encapsulated SCE, and free SCE]. Cell viability was evaluated using MTT assays 24 h, 48 h, and 72 h after MCF-7 cells were grown in 96-well plates. Similarly, MTT experiments revealed that in the niosome-encapsulated SCE group, MCF-7 cell viability was reduced [Fig. 3]. Briefly, cell proliferation of MCF-7 cells was significantly inhibited by niosome-encapsulated SCE compared to the control groups [niosome-mixed SCE and free SCE] [p < 0.01]. The above results indicated that niosome-encapsulated SCE may inhibit MCF-7 breast cancer cell proliferation. However, in the niosome-mixed SCE and free SCE groups, the survival rates of cancer cells after 72 h were 40% and 43%, respectively. Our results confirmed that sea cucumber extract inhibits the proliferation of MCF-7 cancer cells.

**Fig. 4:**
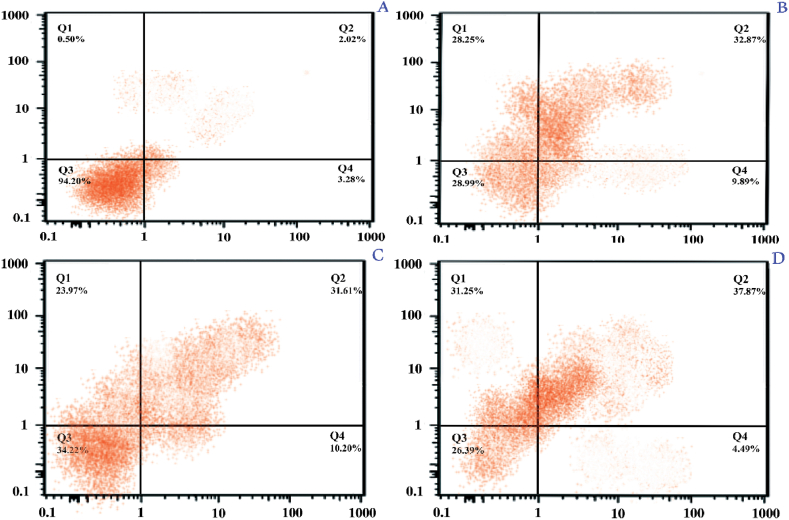
*Sea cucumber extracts* cause MCF-7 cells to undergo apoptosis. Flow cytometry detection of apoptosis in HUVEC normal cells [A], control groups (niosome mixed SCE [B] and free SCE [C] and niosome-encapsulated SCE [D] group of MCF-7 cells. The fraction of apoptotic cells in Q1, Q2, and necrotic cells in Q3 and live cells in Q4 quadrants was calculated using FlowJo software. **P < 0.01.

Flow cytometry was used to determine apoptosis in the niosome-encapsulated SCE group and control groups [niosome mixed SCE with and free SCE] of MCF-7 cells [Fig. 4]. In the niosome-encapsulated SCE group of MCF-7 cell lines, the proportion of apoptotic cells was substantially higher than in the niosome mixed SCE with and free SCE groups. Niosome-encapsulated SCE increased total apoptosis [69.12%] in MCF-7 cells, according to these findings. The proportion of total apoptosis in the two control groups, niosome mixed SCE and free SCE, was 61.12 and 55.58%, respectively.

**Fig. 5:**
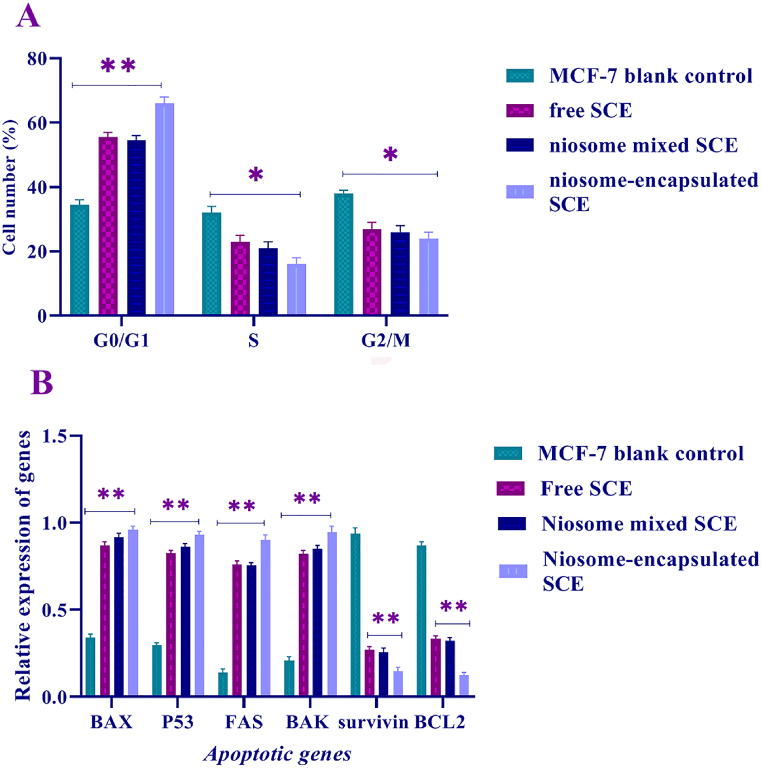
*Sea cucumber extracts* reduce tumor cell proliferation by inducing apoptosis. *Sea cucumber extract* treatment of the MCF-7 cell line led to the induction of the expression of the proapoptotic genes BAX, P53, FAS, and BAK at a significant level **P < 0.001 and a reduction in the expression of the antiapoptotic genes SURVIVIN and BCL2 at a significant level * P < 0.01. Data were normalized by the GAPDH reference gene. There was no significant difference in the expression of pro/anti-apoptotic genes in the normal MCF-7 cells and positive control [blank MCF-7 cells].

Cell cycle advancement is linked to the acceleration of cell proliferation. Flow cytometry was used to examine cell cycle regulation in the niosome-encapsulated SCE group and control groups [niosome mixed SCE and free SCE] of MCF-7 cells [Fig. 5]. *Sea cucumber extracts* in the MCF-7 cells showed an increase in G0/G1 and a decrease in the S phase to G2/M phase ratio compared to the MCF-7 blank control cell. *Sea cucumber extracts* hindered cell cycle progression, according to these findings [Fig. 5A]. For this study, proapoptotic *P53*, *BAK*, *FAS*, and *BAX* genes and antiapoptotic genes *BCL2* and *SURVIVIN* were selected, and real-time PCR was performed to evaluate the expression of these genes in three MCF-7 cell lines of the niosome-encapsulated SCE group and control groups [niosome mixed SCE and free SCE]. As expected, proapoptotic gene expression was significantly higher in the niosome-encapsulated SCE groups than in the niosome mixed SCE and free SCE groups [Fig. 5B, p < 0.001]. We next assessed antiapoptotic gene [*BCL2*, *SURVIVIN*] expression in 3 different MCF-7 cell groups. *BCL2* and *SURVIVIN* gene expression was higher in the 2MCF-7 control cell lines [niosome mixed SCE and free SCE] than in niosome-encapsulated SCE group cells [p < 0.01]. Fig. 5 shows that the expression of *P53*, *BAK*, *BAX*, and *FAS* apoptotic genes increased in the MCF-7 cell line treated with sea cucumber extracts. In contrast, the expression of *BCL2* and *SURVIVIN* antiapoptotic genes showed a decrease in the MCF-7 cell line treated with sea cucumber extracts. As a result, treatment with sea cucumber extracts enhanced the production of apoptotic genes while decreasing the expression of antiapoptotic genes in the MCF-7 cancer cell line. In contrast, when MCF-7 blank cells were compared to cells in the treatment groups, apoptotic gene expression was found to be significantly higher, with a P < 0.001 significance level. These findings show that sea cucumber extract has a direct influence on the activation of apoptosis in this cell line. According to the results of this study, encapsulation of sea cucumber extract with niosome nanoparticles enhances the induction of apoptotic cells, which can be assessed at a significance level of P < 0.05.

## 4 Discussion

Sea cucumber is the most common in the Persian Gulf and is found across southern Iran [28]. Because of their great medicinal and nutritional potential, sea cucumbers are the most marketed and harvested of all echinoderms [29]. Sea cucumbers have been used in traditional medicine in Southeast Asia and the Middle East for many years to treat hypertension, bronchitis, arthritis, wounds, and constipation, among other ailments [30]. Although much research has been done on sea cucumber extracts to find powerful bioactive chemicals with putative anti-inflammatory, immunostimulatory, and anticancer effects, there have been relatively few investigations on sea cucumber bioactivities [31]. Given the huge number of bioactive chemicals found in sea cucumbers [32]. Many investigations on the antibacterial effects of a variety of marine animals, including Echinodermata, have been conducted in recent years by various countries [33].

Sea cucumber exhibited the highest antibacterial activity compared to other marine species, including Porifera, Bryozoa, Molluska, Coral, and Annelida [ringed worms] [34]. Methanol, aqueous methanol, ethanol, and chloroform extracts, as well as triterpene compounds, have been employed in the bulk of research on sea cucumber antibacterial and antifungal properties [8,35]. The antifungal activity of *H. polii* [a particular type of sea cucumber] in the Mediterranean Sea was studied using standard disc diffusion by certain researchers [35,36]. Research demonstrated that 2.5 mg/ml ethanolic extracts of the sea cucumber body wall exhibited significant antifungal activity against *Aspergillus flavus*, *Aspergillus niger*, and *C. albicans* [38]. According to the disk diffusion technique of many studies, the antimicrobial activities of the aqueous methanol extract of H. polii have a significant inhibitory impact on *A. funmigatus* and a lesser ability to inhibit *Trichophyton rubram* but no effect on *C. albicans* [39].

Sea cucumber extract has also been studied for its cancer-fighting properties [40]. The antitumorigenic effects of triterpene glycosides derived from sea cucumbers were investigated in an animal model of S180 sarcoma and mouse Lewis lung cancer cell lines [41]. In human colorectal cancer, an aqueous extract of sea cucumber was found to dramatically suppress proliferation and cause intense cytotoxicity in Caco-2 cells, a kind of intestinal cell [[42], [56]]. In DLD-1, WiDr, and Caco-2 human colon carcinoma cells, extracted sphingoid bases of sea cucumber [Stichopus variegatus] demonstrated strong cytotoxic effects and reduced cell proliferation, as well as induction of apoptosis via caspase 3 activation [43]. Holothurin A [HA] and 24-dehydroechinoside A [DHEA], both sulfated triterpene glycosides derived from the sea cucumber genus [Pearsonothuria graeffei], were found to have antitumor and antimetastatic properties in research [[44], [45], [46], [47], [48], [49], [50]].

Niosomes have been the subject of an increasing number of studies in recent years due to the advancement of nanotechnologies in the pharmaceutics industry. Due to their capacity to encapsulate various medications to boost their stability and effectiveness, niosomes can be used as an alternative to liposomes and polymersomes. In contrast to other nanoparticles, liposomes, polymersomes, and niosomes share a lot of structural commonalities and can all be filled with both hydrophilic and hydrophobic medications. They might therefore co-deliver hydrophilic and hydrophobic medications in the same vesicle. Niosomes have received a lot of attention due to their outstanding biocompatibility and comparatively low toxicity. Niosomes have advantages over liposomes, such as superior stability, low cost, simplicity in formulation, and scaling-up. Because non-ionic surfactants, which are the building blocks of niosomes, are more physically and chemically stable than lipids, niosomes are significantly more stable [53,54].

However, no research on the use of nanomaterials to improve sea cucumber extract has been conducted thus far [[45], [46], [47], [48], [49], [50], [51], [52], [53], [54], [55], [56]]. This is one of the initial studies to investigate encapsulating sea cucumber extract by niosome nanoparticles. The encapsulating technique was used in this investigation after the sea cucumber extract was obtained. Fig. 1 shows a scanning electron microscopic picture of niosomes. Most niosomes have a circular form, with a size distribution of approximately 75.32 ± 0.34 nm or less, according to SEM and TEM examinations, which matches the results of dynamic light scattering experiments. However, the effective encapsulation of the extracts was shown by increasing the size of niosome nanostructures in the SCE encapsulating group [80.46 ± 1.31] while maintaining a homogeneous spherical structure. *S. aureus* and Candida albicans were inhibited by niosome-encapsulated SCE at doses of 100 μg/ml, according to our observations. Our findings show that antibacterial activity can be found in materials containing various components in the sea cucumber body. Furthermore, by encapsulating sea cucumber extract, niosome nanoparticles inhibit the molecular reactivity of the extract components with the growth medium, lowering the quality of the extract. Furthermore, niosomal nanoparticles improve drug delivery. The drug's adverse effects, such as toxicity and hemolysis, are lessened by niosome. Reduced side effects and a sharp rise in breast cancer cells were observed. The P-glycoprotein (P-gp) is efficiently inhibited by niosomes, increasing the bioavailability of anticancer and antiviral medications. To fight various illnesses, several pharmacological substances may be able to use niosomal drug delivery. By overcoming the anatomical barrier of the gastrointestinal system by transcytosis of M cells from Peyer's patches in the intestinal lymphatic tissues, it improves bioavailability. The reticuloendothelial system absorbs the niosomal vesicles. Such localized medication accumulation is employed to treat disorders where some issues infiltrate the liver and spleen cells [[53], [55]].

As a result, sea cucumber extract encapsulated with niosomes dramatically boosted its antibacterial activity. As a result, sea cucumbers encapsulated in niosomes can be used as a source of antibacterial chemicals, making them suitable candidates for the production of pharmaceutical and medicinal compounds, as well as antibiotics. The effect of sea cucumber water extract [Sichopus variegatus] on rat spinal astrocyte cell lines was investigated by a researcher. Extracts (0.1, 1.0, 5.0, and 10.0 μg/ml) were produced. Their findings imply a dose-dependent effect of *S. aureus* water extract on spinal astrocyte proliferation and differentiation. However, using a novel formulation of sea cucumber extract, our recent investigation found that it had superior antibacterial and antifungal properties. On day three after treatment with niosome-encapsulated SCE, MCF-7 cell proliferation was reduced, although normal cell viability was unaffected. By enhancing polarity, the addition of Span 60 to niosomes improved tumor cell specificity. As a result, encapsulating SCE in a niosome increased its anticancer activity in a particular way. Encapsulated sea cucumber extracts inhibited cell progression in MCF-7 cells by increasing G0/G1 and decreasing S phase relative to G2/M phase; as a result, it activated the apoptosis signaling pathway and led to the induction of apoptosis in 69.12% of tumor cells by increasing the expression of proapoptotic genes.

The results indicate that sea cucumber species from the Persian Gulf are a promising source of natural chemicals with anticancer properties, paving the path for novel marine natural products to be discovered. This is the first demonstration that niosome-encapsulated SCE contains antibacterial and anticancer chemicals that, according to their specific characteristics, boost antitumor activity. Further research is needed to purify and characterize the biological activity stated above.

## Ethical approval

The authors of this article confirm that all methods were carried out in accordance with relevant guidelines and regulations. The authors of this article state that all methods are reported in accordance with ARRIVE guidelines (https://arriveguidelines.org). All animal protocols were performed in accordance with the Ethical Committee and Research Deputy of the Islamic Azad University of East-Tehran Branch, Iran for the Care and Use of Laboratory Animals and were approved by the Institutional Animal Care and Use Committee guidelines of Islamic Azad University, East-Tehran, Iran.

## Author contribution statement

Tohid Piri-Gharaghie, Ghazal Ghajari, Neda Jegargoshe-Shirin: Conceived and designed the experiments; Performed the experiments; Analyzed and interpreted the data.

Maryam Hassanpoor: Conceived and designed the experiments; Performed the experiments; Analyzed and interpreted the data; Contributed reagents, materials, analysis tools or data; Wrote the paper.

Ali Farhadi-Biregani, Shahoo Khayati, Amir Mirzaei: Contributed reagents, materials, analysis tools or data; Wrote the paper.

## Funding statement

This research did not receive any specific grant from funding agencies in the public, commercial, or not-for-profit sectors.

## Data availability statement

Data included in article/supp. Material/referenced in article.

## Declaration of interest's statement

The authors declare no conflict of interest.

## References

[1] Abdian N. (2015). Comparison of human dermal fibroblasts (HDFs) growth rate in culture media supplemented with or without basic fibroblast growth factor (bFGF). Cell Tissue Bank..

[2] Geahchan S., Ehrlich H., Rahman M.A. (2021). The anti-viral applications of marine resources for COVID-19 treatment: an overview. Mar. Drugs.

[3] Mondol M.A., Shin H.J., Rahman M.A., Islam M.T. (2017). Sea cucumber glycosides: chemical structures, producing species and important biological properties. Mar. Drugs.

[4] Bordbar S., Anwar F., Saari N. (2011). High-value components and bioactives from sea cucumbers for functional foods—a review. Mar. Drugs.

[5] Singh H., Parida A., Debbarma K., Ray D.P., Banerjee P. (2020). Common marine organisms: a novel source of medicinal compounds. IJBS.

[6] Rotter A., Barbier M., Bertoni F., Bones A.M., Cancela M.L., Carlsson J., Carvalho M.F., Cegłowska M., Chirivella-Martorell J., Conk Dalay M., Cueto M. (2021). The essentials of marine biotechnology. Front. Mar. Sci..

[7] Kalinin V.I. (2021).

[8] Shakouri A., Shoushizadeh M.R., Nematpour F. (2017). Antimicrobial activity of sea cucumber (Stichopus variegatus) body wall extract in Chabahar Bay, Oman Sea. Jundishapur J. Nat. Pharm. Prod..

[9] Purcell S.W., Conand C., Uthicke S., Byrne M. (2016). Ecological roles of exploited sea cucumbers. Oceanogr. Mar. Biol..

[10] Kamyab E., Kellermann M.Y., Kunzmann A., Schupp P.J. (2020). YOUMARES 9-the Oceans: Our Research, Our Future.

[11] Mashjoor S., Yousefzadi M. (2017). Holothurians antifungal and antibacterial activity to human pathogens in the Persian Gulf. J. Mycol. Med..

[12] Ghasemi-Dehkordi P., Allahbakhshian-Farsani M., Abdian N., Mirzaeian A., Saffari-Chaleshtori J., Heybati F., Mardani G., Karimi-Taghanaki A., Doosti A., Jami M.S., Abolhasani M., Hashemzadeh-Chaleshtori M. (2015). Comparison between the cultures of human induced pluripotent stem cells (hiPSCs) on feeder-and serum-free system (Matrigel matrix), MEF and HDF feeder cell lines. J. Cell Commun. Signal..

[13] Abdian N., Ghasemi-Dehkordi P., Hashemzadeh-Chaleshtori M., Ganji-Arjenaki M., Doosti A., Amiri B. (2015). Comparison of human dermal fibroblasts (HDFs) growth rate in culture media supplemented with or without basic fibroblast growth factor (bFGF). Cell Tissue Bank..

[14] Kamyab E., Kellermann M.Y., Kunzmann A., Schupp P.J. (2020). YOUMARES 9-the Oceans: Our Research, Our Future.

[15] Khotimchenko Y. (2018). Pharmacological potential of sea cucumbers. Int. J. Mol. Sci..

[16] Liu Y., Dave D., Trenholm S., Ramakrishnan V.V., Murphy W. (2021). Effect of drying on nutritional composition of atlantic Sea Cucumber (Cucumaria frondosa) viscera derived from newfoundland fisheries. Processes.

[17] Farhood B., Geraily G., Alizadeh A. (2018 Mar). Incidence and mortality of various cancers in Iran and compare to other countries: a review article. Iran. J. Public Health.

[18] Lu R.M., Hwang Y.C., Liu I.J., Lee C.C., Tsai H.Z., Li H.J., Wu H.C. (2020 Dec). Development of therapeutic antibodies for the treatment of diseases. J. Biomed. Sci..

[19] Gurunathan S., Qasim M., Kang M.H., Kim J.H. (2021). Role and therapeutic potential of melatonin in various type of cancers. OncoTargets Ther..

[20] Society A.C. (2019–2021).

[21] Keshavarz M., Shamsizadeh F., Tavakoli A., Baghban N., Khoradmehr A., Kameli A., Rasekh P., Daneshi A., Nabipour I., Vahdat K., Farrokhnia M. (2021). Chemical compositions and experimental and computational modeling activity of sea cucumber Holothuria parva ethanolic extract against herpes simplex virus type 1. Biomed. Pharmacother..

[22] Patra J.K., Das G., Fraceto L.F., Campos E.V., del Pilar Rodriguez-Torres M., Acosta-Torres L.S., Diaz-Torres L.A., Grillo R., Swamy M.K., Sharma S., Habtemariam S. (2018). Nano based drug delivery systems: recent developments and future prospects. J. Nanobiotechnol..

[23] Chenthamara D., Subramaniam S., Ramakrishnan S.G., Krishnaswamy S., Essa M.M., Lin F.H., Qoronfleh M.W. (2019). Therapeutic efficacy of nanoparticles and routes of administration. Biomater. Res..

[24] Piri Gharaghie T. (2022). Seyed Ataollah Sadat Shandiz, and Sheida Beiranvand. Evaluation of silver nanoparticles effects on bla-per1 gene expression for biofilm formation in isolates of antibiotic-resistant Acientobacter Bumanni by real time PCR method. Cell. Mol. Res. (Iran. J. Biol.).

[25] Patra J.K., Das G., Fraceto L.F., Campos E.V., del Pilar Rodriguez-Torres M., Acosta-Torres L.S., Diaz-Torres L.A., Grillo R., Swamy M.K., Sharma S., Habtemariam S. (2018). Nano based drug delivery systems: recent developments and future prospects. J. Nanobiotechnol..

[26] Joshi S., White R., Sahu R., Dennis V.A., Singh S.R. (2020). Comprehensive screening of drug encapsulation and Co-encapsulation into niosomes produced using a microfluidic device. Processes.

[27] Ag Seleci D., Seleci M., Walter J.G., Stahl F., Scheper T. (2016). Niosomes as nanoparticular drug carriers: fundamentals and recent applications. J. Nanomater..

[28] Sharifi M., Yegdaneh A., Sajjadi S.E., Shushizadeh M. (2017). Identification and quantification of Phthalate pollution in Holothuria atra: a sea cucumber from the Persian Gulf (Iran). Jundishapur J. Nat. Pharm. Prod..

[29] Rao N.N., Chowdary P., Divya Y., Laksmi T., Latha K., Sirisha P. (2018). Niosomes: a vesicular drug delivery system. Res. J. Pharm. Technol..

[30] Ebrahimi Hadi, Mohebbi G.H., Vazirizadeh Amir, Nabipour Iraj, Nafisi Bahabadi M. (2015).

[31] Razi NS, Arast Y, Nazemi M, Pourahmad J. The use of methanolic extract of Persian Gulf Sea Cucumber, Holothuria, as potential anti-cancer agents. Int. Pharm. Acta.;1(2):208-217.

[32] Rasyid A., Yasman Y., Putra M.Y. (2021 Jul 23). Current prospects of nutraceutical and pharmaceutical use of sea cucumbers. Pharmacia.

[33] Macedo M.W., Cunha N.B., Carneiro J.A., Costa R.A., Alencar S., Cardoso M.H., Franco O.L., Dias S.C. (2021). Marine organisms as a rich source of biologically active peptides. Front. Mar. Sci..

[34] Datta D., Talapatra S.N., Swarnakar S. (2015). Bioactive compounds from marine invertebrates for potential medicines-an overview. Int. Lett. Nat. Sci..

[35] Ceesay A., Nor Shamsudin M., Aliyu-Paiko M., Ismail I.S., Nazarudin M.F., Mohamed Alipiah N. (2019 Apr 15). Extraction and characterization of organ components of the Malaysian sea cucumber Holothuria leucospilota yielded bioactives exhibiting diverse properties. BioMed Res. Int..

[36] Darya M., Sajjadi M.M., Yousefzadi M., Sourinejad I., Zarei M. (2020 Dec). Antifouling and antibacterial activities of bioactive extracts from different organs of the sea cucumber Holothuria leucospilota. Helgol. Mar. Res..

[37] Omran N.E., Allam N.G. (2013 Nov). Screening of microbial contamination and antimicrobial activity of sea cucumber Holothuria polii. Toxicol. Ind. Health.

[38] Mokhlesi A., Saeidnia S., Gohari A.R., Shahverdi A.R., Nasrolahi A., Farahani F., Khoshnood R., Es' haghi N. (2012). Biological activities of the sea cucumber Holothuria leucospilota. Asian J. Anim. Vet. Adv..

[39] Husni A., Shin I.S., Chung D. (2014). Effect of extraction methods on antifungal activity of sea cucumber (Stichopus japonicus). Agritech.

[40] Janakiram N.B., Mohammed A., Rao C.V. (2015). Sea cucumbers metabolites as potent anticancer agents. Mar. Drugs.

[41] Zhang Y., Yi Y. (2011 Feb 1). Studies on antitumor activities of triterpene glycoside colochiroside A from sea cucumber Colochirus anceps. Zhongguo Zhong yao za zhi= Zhongguo zhongyao zazhi= China J. Chin. Mater. Med..

[42] Karamkhani A., Saki J. (2019 May 31). Assessment of apoptosis induction by methanol extract of Sea Cucumber in blastocystis hominis isolated from human samples using flow cytometry and DNA fragmentation test. Jundishapur J. Nat. Pharm. Prod..

[43] Sugawara T., Zaima N., Yamamoto A., Sakai S., Noguchi R., Hirata T. (2006). Isolation of sphingoid bases of sea cucumber cerebrosides and their cytotoxicity against human colon cancer cells. Biosci., Biotechnol., Biochem..

[44] Zhao Q., Xue Y., Liu Z.D., Li H., Wang J.F., Li Z.J., Wang Y.M., Dong P., Xue C.H. (2010). Differential effects of sulfated triterpene glycosides, holothurin A1, and 24‐dehydroechinoside A, on antimetastasic activity via regulation of the MMP‐9 signal pathway. J. Food Sci..

[45] Senadheera T.R., Dave D., Shahidi F. (2020). Sea cucumber derived type I collagen: a comprehensive review. Mar. Drugs.

[46] Marianecci C., Di Marzio L., Rinaldi F., Celia C., Paolino D., Alhaique F., Carafa M. (2014). Niosomes from 80s to present: the state of the art. Adv. Colloid Interface Sci..

[47] Moghassemi S., Hadjizadeh A. (2014). Nano-niosomes as nanoscale drug delivery systems: an illustrated review. J. Contr. Release.

[48] Sahab-Negah S., Ariakia F., Jalili-Nik M., Afshari A.R., Salehi S., Samini F., Rajabzadeh G., Gorji A. (2020). Curcumin loaded in niosomal nanoparticles improved the anti-tumor effects of free curcumin on glioblastoma stem-like cells: an in vitro study. Mol. Neurobiol..

[49] Zarinnezhad Amineh, Mohamad Hassan Shahhoseini, Tohid Piri Gharaghie (2021). Evaluating the relative frequency of fungal infections in the serum of patients with multiple sclerosis and healthy subjects using PCR. Biol. J. Microorganism.

[50] Piri Gharaghie Tohid (2018). and Seyed Ataollah Sadat Shandiz. "The inhibitory effects of silver nanoparticles on bap gene expression in antibiotic-resistant acientobacter bumanni isolates using real-time PCR. J. Ilam Univ. Med. Sci..

[51] Beiranvand S. (2022). Novel NAD‐independent Avibacterium paragallinarum: Isolation, characterization and molecular identification in Iran. Vet. Med. Sci..

[52] Rezaei H., Zabihzadeh M., Ghorbani M., Goli Ahmadabad F., Mostaghimi H. (2017). Evaluation of dose enhancement in presence of gold nanoparticles in eye brachytherapy by 103Pd source. Australas. Phys. Eng. Sci. Med..

[53] Kazi K.M., Mandal A.S., Biswas N., Guha A., Chatterjee S., Behera M., Kuotsu K. (2010 Oct). Niosome: a future of targeted drug delivery systems. J. Adv. Pharm. Technol. Research (JAPTR).

[54] Ge X., Wei M., He S., Yuan W.E. (2019). Advances of non-ionic surfactant vesicles (niosomes) and their application in drug delivery. Pharmaceutics.

[55] Piri Gharaghie, Tohid, Abbas Doosti (2021). and Seyed Abbas Mirzaei. Prevalence and antibiotic resistance pattern of Acinetobacter spp. infections in Shahrekord medical centers. Dev. Biol..

[56] Piri Gharaghie T. (2020). A review of the epidemiology and clinical signs of SARS-COV-2. NCMB J..

