# Supplementary material for: Investigation of antibacterial and anticancer effects of novel niosomal formulated Persian Gulf Sea cucumber extracts
Source: Heliyon. 2023 Feb 28;9(3):e14149. doi: 10.1016/j.heliyon.2023.e14149 (PMC10018472; doi:10.1016/j.heliyon.2023.e14149)
Supplement: Multimedia component 1 [file mmc1.docx]

**Supplementary materials**

Table S1. Bioactive components detected and measured via GC-MS indifferent solvent extracts of body organs of sea cucumber.

| Compounds | | % of Compounds in extract |
| --- | --- | --- |
| Ketones | 4-hydroxy-4-methyl-2-Pentanone | 27±0.15 |
|  | 4-methyl-3-Penten-2-one | 1.9±0.12 |
| Phenols | 2,4-bis(1,1-dimethylethyl)-Phenol- | 5.9±0.09 |
| Alcohols | 2-Furanmethanol | 4.75±0.16 |
|  | (+)-trans,trans-5-caranol | 4.51±0.17 |
|  | 10-Methyl-8-tetradecen-1-ol-acetate | 1.14±0.11 |
|  | Glycerin | 0.8±0.02 |
| Hydrocarbons | 3-Chlorooctane | 3.37±0.11 |
|  | 3,4-dimethyl-1-decene | 10.5±0.15 |
|  | 3-ethyloctane | 4.02±0.09 |
|  | 1- methyl -3-ethyl Cyclopentane | 5.45±0.13 |
|  | 1-Nonene | 11.7±0.17 |
| Fatty acids | Tetradecanoic acid | 1.35±0.04 |
|  | n-Hexadecanoic acid | 8.8±0.09 |
|  | 9-Hexadecenoic acid | 5.7±0.13 |
|  | Heptadecanoic acid | 3.5±0.08 |
|  | Eicosanoic acid | 0.45±0.03 |
